# A Linear-RBF Multikernel SVM to Classify Big Text Corpora

**DOI:** 10.1155/2015/878291

**Published:** 2015-03-23

**Authors:** R. Romero, E. L. Iglesias, L. Borrajo

**Affiliations:** Department of Computer Science, Higher Technical School of Computer Engineering, University of Vigo, 32004 Ourense, Spain

## Abstract

Support vector machine (SVM) is a powerful technique for classification. However, SVM is not suitable for classification of large datasets or text corpora, because the training complexity of SVMs is highly dependent on the input size. Recent developments in the literature on the SVM and other kernel methods emphasize the need to consider multiple kernels or parameterizations of kernels because they provide greater flexibility. This paper shows a multikernel SVM to manage highly dimensional data, providing an automatic parameterization with low computational cost and improving results against SVMs parameterized under a brute-force search. The model consists in spreading the dataset into cohesive term slices (clusters) to construct a defined structure (multikernel). The new approach is tested on different text corpora. Experimental results show that the new classifier has good accuracy compared with the classic SVM, while the training is significantly faster than several other SVM classifiers.

## 1. Introduction

The amount of information stored in public resources continues to grow. For example, the Medline bibliographic database, the most important source in the biomedical domain, stores documents since 1950 and contains more than 22 million citations. Thus, in order to manage this volume of documents, the use of sophisticated computer tools must be considered.

In the last years, researchers show a special interest of applying text mining techniques to the field of biomedicine, as pattern recognition, automatic categorization, or classification techniques. In order to get good results, the need to establish a unified data structure to represent documents must be accomplished.

A well-known data structure supported by the scientific community is the* sparse matrix* [1], which is commonly managed by classifiers as input data. In it, each document is decomposed as a vector of its more relevant terms (words).

Unfortunately, although an efficient data structure solves problems related to performance, other inconveniences about the size of the corpora impact negatively over classifiers and their accuracy. Data imbalance problems exist in a broad range of experimental data and have captured the attention of researchers [2, 3]. Data imbalance occurs when the majority class in a document corpus is represented by a large portion of documents, while the minority class has only a small percentage [4]. When a text classifier encounters an imbalanced document corpus, the performance of machine learning algorithms often decreases [5–8].

Another important situation in a classification process, which can render the problem unmanageable, is related to the sparse matrix dimensionality. The matrix dimension is directly connected to the amount of attributes (terms) of the documents included in it, affecting the performance of the classifier and attaching a high computational cost. At this point, algorithms to select relevant terms from whole data structure must be considered. As a result, an optimized sparse matrix is generated.

Regarding classifiers, support vector machine (SVM) [9–12] is one of the most well-known classification techniques used within the scientific community. It obtains good results in a variety of classification problems, although it is difficult to determine its parameterization with imbalanced data. A SVM classifier uses a kernel function to make a transformation over the data and change the workspace, separating relevant from nonrelevant documents. Taking into account that some kernels have additional parameters that must be selected, the parameterization of a SVM has a high cost.

As with other classifiers, SVMs are not suitable to classify large datasets due to their high training complexity. Support vectors are internally computed to represent the dataset; this helps to find a hyperplane that separates the contents of each class. The complexity of a SVM is given by the number of support vectors needed to get the hyperplane. Data dimensionality negatively affects the kernel coverage, such that a unique kernel may not be enough to get an optimal division between classes.

One solution is to divide the dataset into small portions, attaching a specific kernel to each slice, decreasing the training complexity, and improving classification results. This idea is known as a* SVM based on a multikernel transformation*.

Multikernel algorithms combine predefined kernels in order to obtain accurate hyperplanes. These kernels and their parametrization are usually determined by different learning methods. However there is not an efficient learning method to cover all classification scenarios, because it is highly dependent of the field of study. Gönen and Alpaydın [14] establish a category of existing multikernel algorithms focused on their learning methods and properties.

- Fixed rules are functions which combine multiple single kernels grouping them as sums or products and working over the data slice by slice [15, 16]. Kernels are usually unweighted and do not need any training before applying them. However, other approximations include several coefficients to weigh each term in order to penalize some multikernel parts. Even so, value coefficients are adjusted based on empirical results or brute-force algorithms.
- Heuristic approaches combine the idea behind fixed rules to weigh each multikernel term under best coefficient values [17, 18]. These values are usually determined by unsupervised algorithms such as ID3 trees, hierarchical clustering, or self-organizing maps, among others, which may be applied separately (term by term) or one over all of them. In almost all cases, the search space is extremely wide (original or feature), becoming the scenario in a NP-complete problem. Thus, the computational cost and the system performance must be taken into account.
- Optimization approaches consist in providing optimal values for kernel function parameters. Usually, based on external models, this optimization can be integrated as a part of a kernel-based learner or reformulated as a different mathematical model for obtaining the parameter values, and then parametrize the learner [19, 20].
- In Bayesian approaches, kernels are combined and interpreted as probabilistic variables. These parameters (in kernels) are used to perform inference for learning them and the base learner parameters. Bayesian functions measure the quality of the resulting kernel function constructed from candidate kernels using a Bayesian formulation. In general, we use as target function the likelihood or the posterior to find the maximum likelihood estimator and then obtain the model parameter values [21, 22].
- Boosting approaches, inspired on ensemble algorithms, combine weak learning models to produce a new complex strong one [23]. A set of pairwise SVM-kernels may be configured and trained separately to get a final voting result in testing stage. There are different ways in which the combination can be done, including the previous approaches. The models may be predefined or it is possible to add a new kernel until the performance stops improving [23, 24].

In this paper, we show a multikernel SVM to manage highly dimensional data, providing an automatic parameterization with low computational cost and improving results against SVMs parameterized under a brute-force search.

The remainder of the paper proceeds as follows. The general text classification model is described in Section 2. The proposed model is presented in Section 3, matching and explaining differences with the previous section. The analysis of experimental tests and comparative results with other authors are shown in Section 4. Finally, the most relevant conclusions are collected at Section 5.

## 2. Text Classification

Text classification is focused on assigning a class to each document of a corpus. Thus, a class encloses those documents which are representative from a specific topic. The class assignment can be performed manually or automatically.

**Figure 1:**
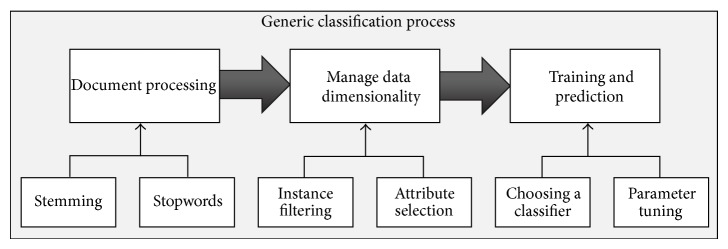
Schema of a generic text classification model.

In general, the text classification process includes a set of steps, as shown in Figure 1. These steps are detailed in the next subsections.

### 2.1. Document Processing

During the first step, each document *d*_*j*_ in the corpus is processed to extract its most representative keywords (terms). As each term *t*_*i*_ has a different relevance when it is used to describe the document content, a numerical weight *w*_*ij*_ is assigned. This weight quantifies the importance of the term for describing the document semantic. Moreover, a data normalizing process is used to transform term weights into a new unified value range, with TF-IDF (term frequency-inverse document frequency) being the most used normalization process [1].

As a result, each document *d*_*j*_ is represented by a *m*-dimensional vector (instance), where *m* is the total number of terms in the corpus and an associated class (relevant or nonrelevant) (see (1) (term vectors for a document corpus)). The similarity between two documents is computed based on the distance of their representative vectors. Consider

(1) t1t2⋯tmclassd1w11w21⋯wm1relevantd2w12w22⋯wm2unrelevant⋮⋮⋱⋮⋮d5w15w25⋯wm5unrelevant⋮⋮⋱⋮⋮dnw1jw2j⋯wmnrelevant.

#### 2.1.1. Stemming and Stopwords

In many cases, irrelevant terms are included on the sparse matrix, thus decreasing the classification results. In order to partially remove the noise, some stemming techniques and stopword removal are used.

Stemming techniques [25] morphologically identify terms and their variants (nouns, adjectives, adverbs, etc.) and reduce the data dimensionality through a step called conflation. It is to extract the stem of all the terms and apply a matching process to fuse or combine the terms, avoiding variants in the final representation.

Stopword lists [26] are wordlists composed of irrelevant terms such as articles, determiners, or interrogative particles. These terms are usually excluded during the document matrix generation.

In this way, combining stopword filtering and stemming techniques helps to avoid nonuseful terms and to significantly improve the information retrieval systems and their results.

### 2.2. Manage Data Dimensionality

In general, the use of stemming and stopword removal is not enough to obtain a good document classification for huge datasets. Thus, in a postprocessing step, algorithms and techniques are focused on reducing, compacting, or transforming the matrix containment. Normally, two approaches are considered.

- Instance filtering focused on balancing the number of instances (documents) in each class (topic), taking into account their difference factor. In some cases, unbalanced problems may negatively affect the classification process causing overfitted models.
- Attribute (term) selection algorithms transform and remove (in some cases) current terms in the document matrix in order to reduce its size and computational cost.

#### 2.2.1. Instance Filtering

Data imbalance problem appears when a majority class, usually the negative class, contains many more instances that the other class [2–4]. When a text classifier encounters an imbalanced document corpus, the machine learning performance often decreases [5–8].

Instance filtering represents a powerful tool against overfitting cases with regard to a specific class type (majority class in almost all cases). Two well-known techniques,* oversampling* and* subsampling*, may be applied on texts to redistribute each class and solve the imbalance [27, 28].

The* subsampling* technique removes instances in the majority class by taking into account a difference factor with the minority class. A random algorithm is usually used to select which instance is removed until the redistribution factor is reached. Similarly, the* oversampling* technique adds new or replicated instances in the minority class until the difference factor with the majority class is reached. Equal to subsampling, a random algorithm is used to select which instances are the base of the replication process.

Finally, both techniques can be applied simultaneously, increasing instances at the minority class and decreasing on the majority class. This process is known as* resampling* [29]. The Weka library [30], used in this study, provides algorithms which implement these techniques.

#### 2.2.2. Attribute Selection

Data sizes can be optimized by trying to find the most relevant attributes (terms) in a dataset. Attribute selection algorithms are focused on the relevance of a term in a document, class, or both, removing, merging, and/or transforming those terms that are less important and generating a new dataset. Therefore, an attribute selection task pursues the following goals: (i) to reach better classification results, (ii) to generate more efficient models, and (iii) to reduce the data dimensionality and therefore computational costs.

The Weka library provides algorithms for the attribute selection. Some of them were previously analyzed by the authors [31]. In this work, we apply the principal component analysis (PCA) algorithm [32]. PCA looks for linear combinations between attributes to remove their individual dependency (noise) and to reduce the original data.

### 2.3. Train and Prediction

Once the document matrix is built and optimized, it can be used as input in a classifier. Train and prediction are divided in two complex steps: (i) choosing a classifier, in which a model must be selected, trained, and tested, and (ii) parameter tuning, involving algorithms and techniques in order to fit the classifier parameters and obtain better results.

#### 2.3.1. Choosing a Classifier

In the last step of the process, a reasoning model is selected to classify those documents contained in the dataset as relevant and nonrelevant.

To perform this task, several algorithms supported by the scientist community were analyzed: *K*-nearest neighbor [4, 7], naive Bayes [7, 33], and SVM [6, 34]. Finally, we choose the SVM classifier because it gets the best results with regard to the text classification [7, 35, 36].

#### 2.3.2. Understanding the Support Vector Machines

SVMs were developed from the theory of statistical learning and structural risk minimization [12, 37]. In almost all cases, linear or nonlinear, a new decision surface is calculated, mapping the input space through a *ϕ* function in which samples are separable. Thus, the idea behind SVMs consists of discovering a hyperplane to discriminate positive and negative samples (relevant and nonrelevant documents).

To understand how it works, consider a separable training set in the input space equal to the identity function (linear case), *S* = {(*d*_*i*_, *y*_*i*_)}_*i*=1_^*n*^ with *d*_*i*_ ∈ ℜ^*m*^ and *y*_*i*_ ∈ {−1,1}, and a linear decision function *f*(*d*) = 〈*w*, *d*〉 + *b*, enclosed by support vectors defining the maximum margin between positive and negative samples, where *b* is the bias hyperplane off-set determined by Karush-Kuhn-Tucker conditions.

In order to get an optimal hyperplane, a quadratic programming optimization must be considered:

(2) minw,ξ: 12wTw+C∑i=1nξi,subject:  yi(wTϕ(di)+b)≥1−ξi,    ξi≥0,

where *ξ*_*i*_ is a slack variable (computed during optimization) which serves to control training errors and keep constraints up, *C* is the trade-off parameter for controlling the compromise between the margin maximization and violated restrictions (soft-margin), and *ϕ*(*d*_*i*_) are the equation coefficients. The class *y*_*i*_ for a document *d*_*i*_ is determined by the sign of (3), where the *sv* parameter is the number of support vectors previously calculated on (2). Consider

(3) $$\begin{array}{c} y_{i}=\mathrm{sign}\overset{sv}{\underset{j=1}{\sum }}\left[\alpha_{j}y_{j}\langle d_{j},d_{i}\rangle +b\right]\\ \mathrm{sign}\left(y_{i}\right)=\left\{\begin{array}{cc} \text{relevant} & \text{if}\;y_{i}\geq 0,\\ \text{unrelevant} & \text{if}\;y_{i}<0. \end{array}\right. \end{array}$$

**Figure 2:**
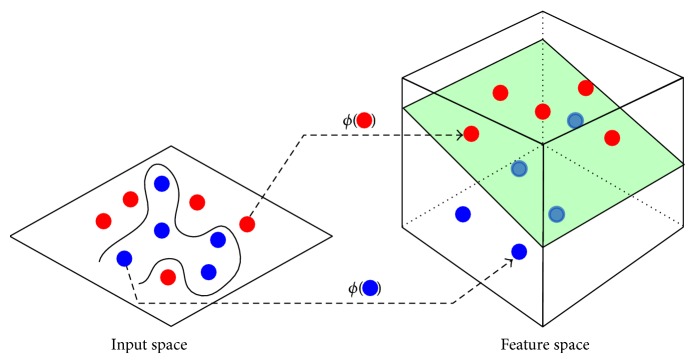
Mapping samples from input to feature space. Image adapted from http://mechamind.in/, verified at 10/29/2014.

For nonlinear cases (*ϕ* is not trivial), input samples are mapped to a feature space, dimensionally higher than original one, where a linear separation may be feasible (see Figure 2). The mapping process is achieved by applying a nonlinear kernel function over each pair of vectors.

As a result, a linear solution is discovered by getting the optimal hyperplane and solving the mentioned nonlinear case [38].

However, given the possible scenario about infinite dimensions in kernel space, the nonlinear mapping function *ϕ* : ℜ^*d*^ → *ħ* cannot be formulated explicitly. A solution consists of expressing the matrix operations in the kernel space *ϕ*(*d*_*i*_)^*T*^ *ϕ*(*d*_*j*_) as dot products in the input space *K*(*d*_*i*_, *d*_*j*_), so-called* kernel trick* [37]. Therefore, (3) is reformulated as follows to include the kernel mapping:

(4) $$\begin{array}{c} y_{i}=\mathrm{sign}\overset{sv}{\underset{j=1}{\sum }}\left[\alpha_{j}y_{j}K\left(d_{j},d_{i}\right)+b\right]. \end{array}$$

#### 2.3.3. Parameter Tuning

The classification process based on SVM is usually supported by several kernels [9]: linear (5), radial basis function (RBF) (6) or Sigmoid (7):

(5) $$\begin{array}{c} \text{Linear:}\;K\left(d_{i},d_{j}\right)=\langle d_{i},d_{j}\rangle , \end{array}$$

(6) Radial  Basis  Function  RBF:    K(di,dj)=exp⁡−σ·di−dj2,

(7) $$\begin{array}{c} \text{Sigmoid:}\;K\left(d_{i},d_{j}\right)=\tanh\left(\sigma \cdot \langle d_{i},d_{j}\rangle +\text{coef}\;\right). \end{array}$$

Some kernel functions, such as radial or sigmoid, provide extra parameters to improve their transformation surface making it more suitable for the dataset morphology. Unfortunately, getting an optimal configuration for these parameters usually results in an NP-complete problem, requiring additional computation cost. Well-known solutions, restricted in almost all cases to a range of values, may be a brute-force search, heuristic methods, or genetic algorithms [35, 36].

#### 2.3.4. Kernel Behavior

**Figure 3:**
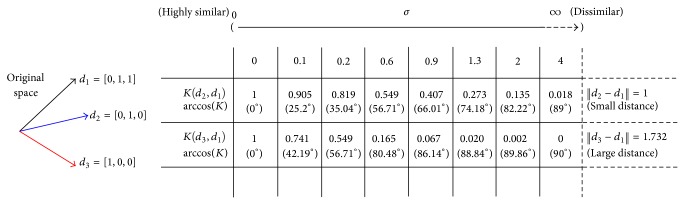
RBF behavior.

In order to understand which range of values are the most suitable to each case, the kernel behavior must be analyzed. As an example, an explanation about the behavior of the RBF kernel is considered here.  Figure 3 describes how to measure the similarity between three vectors *d*_1_ = [0,1, 1], *d*_2_ = [0,1, 0], and *d*_3_ = [1,0, 0] using a RBF kernel, in which *d*_1_ − *d*_2_ are more similar than *d*_1_ − *d*_3_. Sigma values and the search space were obtained based on practical guides and empirical tests [9, 36, 39, 40].

In practice, distances between samples are estimated using the same *σ* value for all cases and must be carefully selected.

**Figure 4:**
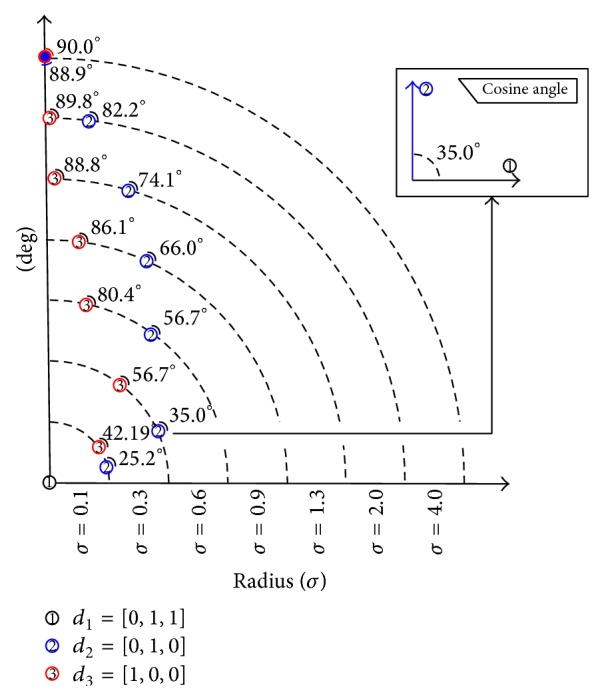
Sigma curves for dissimilar documents.

- Kernel values close to 1 mean that samples are in the same class. Otherwise, values close to 0 mean that samples are in different classes.
- If cosine values are close to 1, samples are very similar in the feature space. Otherwise, if cosine values are close to 0, samples are very dissimilar in the feature space.
- if *σ* values are gradually increased, the angle between vectors {*d*_*j*_, *d*_*k*_} denotes that they are closer to each other, in the feature space, than other ones like {*d*_*i*_, *d*_*k*_}. Therefore if values are increased, the first angle will increase less than the second one (see Figure 4).

Following the previous criteria, an intermediate value represents the best choice to compute the similarity for all vectors. However, in our tests 0.2 and 0.6 values were considered because they maintain both the smallest distance for *d*_1_, *d*_2_ and the largest for *d*_1_, *d*_3_ at the same time.

## 3. Proposed Classification Model

In this section, we introduce a novel text classifier based on SVM over a set of modified RBF kernels. It is developed to manage highly unbalanced data, to autoparameterize itself under low computational cost, and to improve results against brute-force search.

The idea behind the model consists of spreading the dataset into cohesive term slices (clusters) to construct a defined structure. Each cluster is attached with an RBF kernel and the remaining (terms not considered for clustering) are enclosed in a linear kernel creating a multikernel model.

The model was developed to solve cases in which a dataset contains very similar samples for both classes, such as scientific corpora, making it difficult to obtain good results using conventional kernels on SVM classifiers.

**Figure 5:**
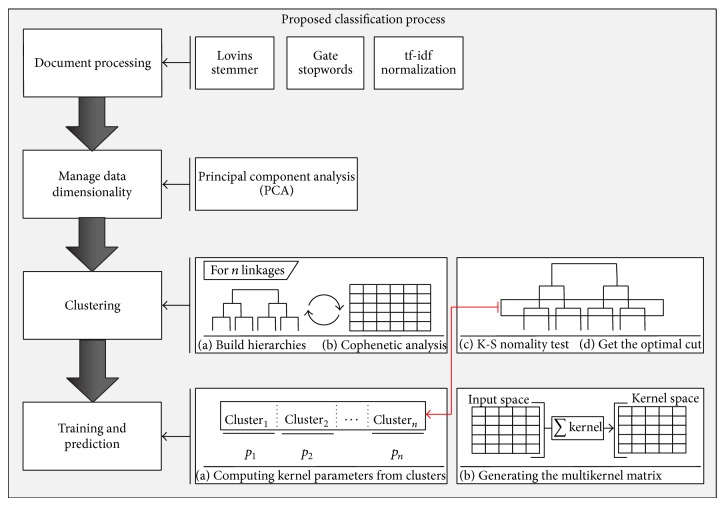
Proposed model architecture.

Figure 5 shows a brief schema of the architecture.

1. For the first step, we use a dataset to generate a document matrix using the vector model. To identify the most relevant terms, a stemming algorithm (Lovins stemmer [25]) and a stopword list extracted from GATE tool [41] are used. In addition, the TF-IDF normalization is used to weight terms based on their frequencies.
2. For the second step, data dimensionality is managed through the principal component analysis (PCA). Terms are compacted using linear combinations between them. As a result, a matrix containing the new subset is generated.
3. The third step starts by transposing the term matrix to build a hierarchical clustering per each linkage method available (see [37, 42] for more details about hierarchical clustering). It continues analyzing resultant hierarchies with the cophenetic matrix to determine which hierarchy best fits the matrix. Finally, the Kolmogorof-Smirnov normality test [43] is applied to each cluster and hierarchy level to determine the optimal cut.
4. The last step consists of building the multikernel Linear-RBF (MLRBF) in the existent clusters from the optimal cut.

All these steps are optimized in order to reduce the computational cost and improve the results.

The next subsections explain each step of the process. The matrix generation is omitted or simply referenced.

### 3.1. Principal Component Analysis

The principal component analysis (PCA) [32] is usually used on text mining to reduce the data dimensionality with a minimum risk of information loss. Dimensionality reduction is accomplished by choosing the eigenvectors, which contain a certain percentage of variance (based on their eigenvalues) with respect to the original data and transforming them.

As a result, the document matrix is reduced according to the linear combination of the most representative terms (the most dispersed), transforming the input space into a smaller one. The new terms are known as principal components.

In this paper, PCA is not used as data reduction algorithm. The internal process looks for linear combinations between terms, producing components which may follow a normal distribution. This means, for relevant documents, that there are terms which are approximately normally distributed. However, this is not the case in the irrelevant documents.

**Figure 6:**
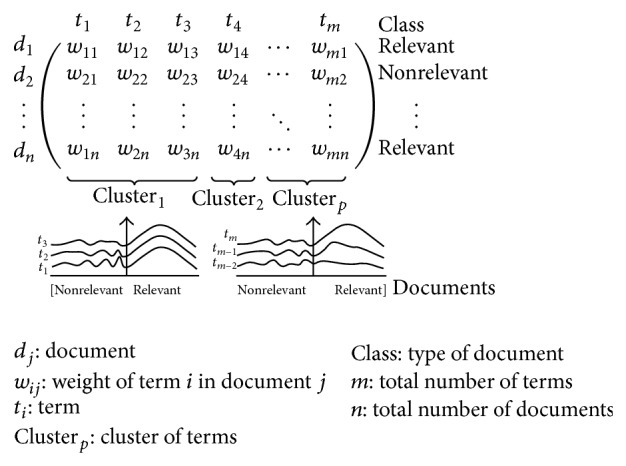
Terms following a normal distribution and agglomerated in cohesive groups.

As a consequence, terms may be agglomerated into cohesive groups (clusters) causing the matrix fragmentation (Figure 6), allowing a better adjustment in the next steps.

The steps to perform a PCA are listed below.

(1) Terms {*t*_1_, *t*_2_, *t*_3_ …, *t*_*m*_} are standardized (zero mean and unit variance, see the following equation) to ensure the independence of each resultant component:
  (8) $$\begin{array}{c} \text{Std}\left(w_{ij}\right)=\frac{w_{ij}-\mu_{i}}{\sigma_{i}}, \end{array}$$
 where *μ*_*i*_ is arithmetic mean of term *i* and *σ*_*i*_: standard deviation of the frequencies of the term *i* in the corpus.
(2) Once the terms are standardized, a correlation matrix is computed and the eigenvalues and eigenvectors are obtained.
(3) Terms are sorted in descending order taking into account their eigenvalues.
(4) Terms with a variance (eigenvalue) lower than 90% are discarded [44].
(5) Terms not discarded (principal components) are used to transform the input space based on their eigenvectors.

### 3.2. Clustering

To divide the matrix in cohesive parts several agglomeration (clustering) techniques have been analyzed.

Well-known solutions such as *K*-means [45] or COWEB [42] were discarded due to their initial parameterization. Hierarchical techniques [37] are the most adequate for our problem because they are not subject to initial requirements for clustering morphology, making it possible to analyze the clusters to determine which size and number are best for an optimal process.

The following subsection helps to get a better understanding of hierarchical clustering and the algorithms that have been developed to analyze the output.

#### 3.2.1. Hierarchical Clustering

**Figure 7:**
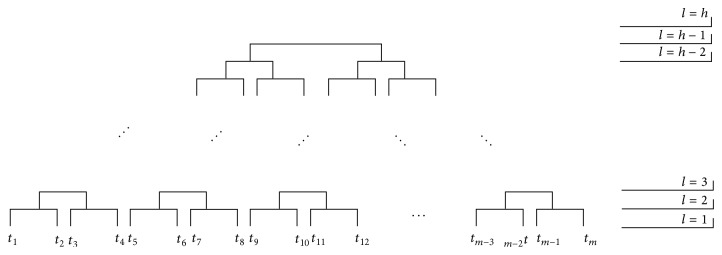
Hierarchical clustering dendogram.

On the hierarchical clustering, entities are agglomerated into groups (clusters) and hierarchically ordered as a heap structure (see Figure 7). Each upper level on the structure contains more components than the previous one due the fact that the clusters are fused into new ones, thus increasing their size. To determine which clusters are the most suitable, each level structure is usually analyzed by algorithms that achieve the* optimal cut*.

Each fusion level is determined by a linkage algorithm which selects the most suitable clusters. Special care is taken in choosing an appropriate linkage method, since it directly affects the final cluster set. In order to do so, a correlation analysis between the original dataset and the resultant hierarchical clustering is performed through the calculation of the cophenetic coefficient [46]. Thus, all linkage methods may be measured to obtain the best one from them.

Several linkage methods such as complete, single, average, median, or ward [47] were tested. The complete linkage (see (9)) is the most suitable method for our datasets. The search space for the complete linkage is focused on far clusters attempting to avoid the local minima problem. Consider

(9) $$\begin{array}{c} \text{distance}\left(A,B\right)\text{:}\;\underset{t_{i}\in A,t_{j}\in B}{\max}\mathrm{dist}\left(t_{i},t_{j}\right), \end{array}$$

where dist⁡(*t*_*i*_, *t*_*j*_) is the distance between the terms *t*_*i*_ and *t*_*j*_ and *A*, *B* are term clusters.

A linkage algorithm also needs a metric to measure distances between terms. In this paper,* Euclidean distance* $\mathrm{dist}(t_{i},t_{j})=\sqrt{\sum_{k=1}^{r}{\left(t_{i}[w_{ki}]-t_{j}[w_{kj}]\right)}^{2}}$ [47] is selected based on empirical results.

Regarding software, a hierarchical clustering algorithm was implemented (see Algorithm 1) in order to include a cophenetic analysis to determine the best linkage method or to calculate the optimal cut. The algorithm iterates over each linkage method computing its associated hierarchy and comparing the correlation through a cophenetic analysis. Once the best linkage method has been identified, its resultant hierarchy is returned.

This implementation includes some operations such as an improved internal distance matrix structure (line 2), logic to restore the distance matrix (line 11), or manageable structures to store each fused level of the hierarchy. These basic operations were crucial to construct a competitive system, reducing the elapsed time needed to build the model.

#### 3.2.2. Optimal Cutting Selection

Once the hierarchy is built, it is analyzed in order to determine the optimal level of the final agglomeration.

Although several well-known solutions [47] may be adapted to produce a feasible solution, some requirements about cluster morphology are not totally satisfied.

- A final cohesive slice is considered as a normal multivariate cluster if all components have a normal distribution.
- An optimal level is only composed of final cohesive slices.
- Large clusters have a smaller probability of following a multivariate distribution than small ones.
- The size and number of the final clusters must be controlled.
- An optimal cutting composed of very small clusters provides a better adjustment of the results (overfitting problem) but increases computational costs. On the contrary, taking only large clusters into account, computational costs are amenably reduced but result in a poorer fit.

Therefore, we present a new algorithm to divide the matrix into cohesive slices and support these requirements (see Algorithm 2).

To evaluate each cluster, a* Kolmogorof-Smirnov test with Lilliefors correction* [43] was used. Thus, each term in a cluster is checked for a normal distribution, assuming that a multivariate cluster is one in which each term is considered normally distributed (*P* value greater than 0.05) [44]. In other words, there is no evidence to reject the null hypothesis per term and therefore that cluster may be considered as a normal multivariate distribution.

### 3.3. Training and Prediction

This last step is focused on parameterizing each kernel portion for training and prediction scenarios.

Our implementation was built on the* LibSVM* [9] library. Several parameters were set by taking into account the* LibSVM* practical guide [40]. The cost parameter *C* was established to 1 according to a small margin, minimizing the trade-off between wrong classified samples.

On the other hand, the kernel (10), defined as a RBF and linear kernels composition (see Section 2.3) was parameterized based on the resultant clusters from the optimal hierarchy level:

(10) MLRBFdi,dj=dita⋯tb,djta⋯tb︸Linear  kernel+∑k=1pRBFkditk1⋯tkl,djtk1⋯tkl,

where *p* = number of clusters in optimal level, RBF = RBF kernel function, [*t*_*k*1_ ⋯ *t*_*kl*_] = terms of the cluster *k*, and [*t*_*a*_ ⋯ *t*_*b*_] = terms nongrouped in any cluster.

**Figure 8:**
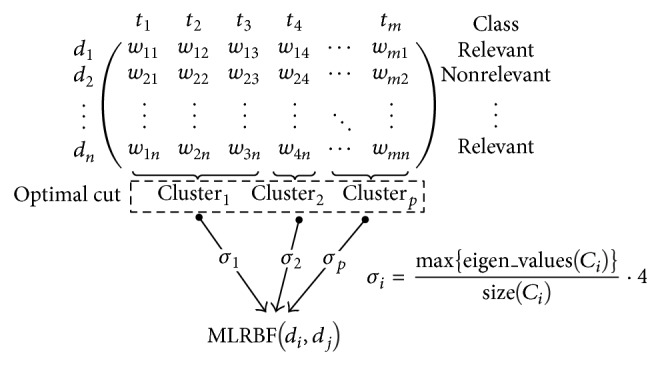
Computing multikernel from optimal level.

Recalling the main idea about identifying cohesive slices to divide the matrix, each slice (cluster) is composed of normal distribution terms and defined as a common pattern (multivariate normal distribution). Clusters provide a simple way to parameterize RBF kernels in the proposed multikernel (see Figure 8).

Each *σ*_*i*_ parameter corresponding to a RBF kernel is computed taking into account the maximum eigenvalue of its associated cluster; specifically, for each term we compute its eigenvalue to finally obtain the maximum by cluster (see (11)). Note that all resultant values have been normalized to prevent absolute eigenvalues, by enclosing them in a defined range interval (0,4] according the explanation in Section 2.3, to get better results. Consider

(11) $$\begin{array}{c} \sigma_{k}=\frac{\max\left\{\text{eigen}\_\text{values}\left(C_{k}\right)\right\}}{\text{size}\left(C_{k}\right)}\cdot 4. \end{array}$$

On the other hand, those terms which are not included in a cluster are enclosed by forming the linear kernel.

In addition, some changes in the input data were performed in order to precompute the multikernel output matrix due to the complexity of the proposed model.

**Figure 9:**
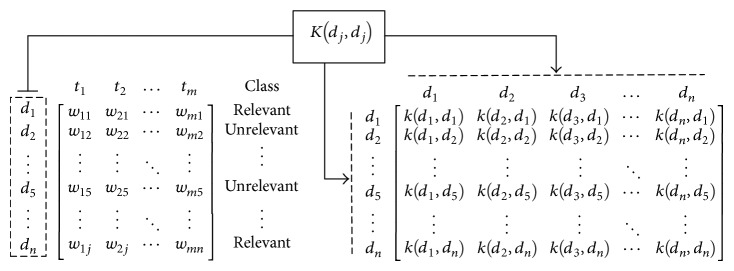
Building the kernel matrix.

In the training, each member in the matrix kernel is computed by applying the MLRBF function over all document vectors. However, the testing step differs in that each test sample is computed against each train vector. To illustrate it, Figure 9 shows how to compute the kernel matrix in a training scenario.

## 4. Results and Discussion

This section includes several tests of preconfigured SVM classifiers and the proposed multikernel from different viewpoints: classification results, model building time performance, and a comparative with other authors.

To evaluate the effectiveness of the model, some statistical measures were used: recall (fraction of relevant documents that are correctly classified), precision (fraction of documents correctly classified as relevant), *F*-measure (harmonic mean between recall and precision), and kappa statistic (which takes the output confusion matrix of an evaluation and reduces it to one value).

As input dataset, the TREC Genomics 2005 corpus [13] was chosen due to the similarity between relevant and nonrelevant documents, offering more realistic classification scenarios. In 2005, the TREC committee provided a set of evaluation tasks to obtain valuable knowledge in biological fields by applying information extraction techniques.

The track is divided in two tasks. One of them consists in categorizing documents regarding different criteria (allele, expression, gene ontology annotation, and tumor) in the genomics domain. Thus, an ad hoc collection, extracted from 4.591.008 MEDLINE records, was prepared by experts to support the task [13]. Having four criteria, resultant records were reorganized to generate each respective corpus (see Table 1).

According to the imbalance problem, only allele and go annotation (GoA) corpora contain enough documents per class to perform our tests applying the subsampling technique. expression and tumor corpuses contain few relevant documents and the oversampling techniques or similar are needed, as used in [13].

Documents were processed to get a suitable structure for SVM classifiers (see Section 3). In addition, PCA was used to determine linear combinations between terms, reducing their amount considerably, and a random subsampling technique to filter instances. Randomly generated subsets contain a uniform distribution (1 : 1), that is, the same number of documents per class. As shown below, it produces different results because samples are not removed in the same order each time they are applied. Thereby, we generated 10 datasets per each corpus (allele and GoA) in order to get trusted results.

Regarding to the parameterization, our multikernel contains internal procedures to determine the most suitable value for each kernel parameter during the classification process (see previous sections). As preconfigured classifiers do not provide automatic methods to get a suitable configuration, sigmoid and RBF parameters (gamma and sigma, resp.) were determined by brute-force. Brute-force was implemented as a grid search, included in the LibSVM library [40], under a set of predefined range values {0.03125,0.0625,0.125,0.25,0.5,1.0,2.0,4.0}. Gamma values (sigmoid case) were reduced to [0,4] taking into account the performed empirical tests. In addition, cost SVM parameter was set to 1 for MLRBF case, to obtain a small-margin hyperplane due to its high precision. On the preconfigured kernels it was determined by the previous grid-search algorithm under a soft-margin range [2^−5^,2^5^].

Table 2 shows a comparison between preconfigured classifiers and our model. Precision, recall, kappa, and *F*-measure statistics were considered to measure the quality of each model. Results were grouped by their minimum (Min), maximum (Max), and average (Avg) values due to the amount of test cases per classifier.

Tests were performed in an* Intel Core i7 at 3.8 Ghz with 8 Gb of RAM*. They were restricted to one execution thread because preconfigured classifiers on LibSVM [9] are implemented under a single execution thread.

With the results, we can conclude that the multikernel obtains competitive values on both corpuses, and it presents a steady behavior in almost all situations compared to other classifiers. The average statistic shows which multikernel obtained the most stable results. As the subsampling technique produces random datasets, if the instances are easily separable by single kernels similar results to our proposed multikernel may be obtained. However, if the instances of different classes are too close, a hyperplane may be extremely difficult to trace since it has only one kernel. Thus, best results on average statistics like recall (0.823), precision (0.893), or *F*-measure (0.858) were achieved by our model on both corpuses.

Table 2 also helps to identify which kernels got maximum or poor results by statistic. Concerning to the allele case, the Sigmoid kernel got some maximum results on the *F*-measure (0.888) or recall (0.882) with gamma values close to zero, but its average or minimum stats are lower than those obtained by the multikernel. The behavior of the linear kernel was very similar to our model, obtaining a maximum peak on precision (0.913). Finally, the RBF got the worst results (sigma values close to 0).

Regarding the GoA corpus, best results were achieved by MLRBF in almost all cases, except recall (Max case), demonstrating that, on those corpuses which are not easily separable on original space (linear case), our multikernel offers results with high values on precision and recall statistics, as a consequence of a very accurate hyperplane. We would like to clarify that the RBF kernel got a high value on the recall statistic at the expense of other stats, achieving poor results on the kappa statistic, meaning that classes were not correctly trained. Therefore, we can conclude that the RBF kernel is not suitable for the GoA corpus.

Table 3 shows the time to build a model respect a specific corpus, divided by folds.

As shown, the building time on linear and MLRBF kernels presents a similar steady growth because the parametrization time of our model is almost constant and linear kernels do not have parameters. Even so, the needed time to configure a linear-SVM is around 1/3 less than MLRBF with regard to allele and a bit less on GoA.

On the other hand, RBF and Sigmoid kernels enclose a high runtime cost because their parameterizations are determined by an external technique. Note that the brute-force cost increases depending on the search space size.

To conclude, the MLRBF kernel offers a good solution with low runtime cost compared with RBF or Sigmoid kernels, but the linear still continues getting the lowest cost.

As a final analysis, a comparison with other authors is included. The TREC 2005 Overview [13] document offers a wide explanation about tasks, tests, and results performed in the competition. Note that the categorization task is focused on how to maximize the number of relevant documents that are correctly classified (recall), putting aside other stats like precision or *F*-measure. In order to compare our system against other authors we reorganized the results based on the *F*-measure (*F*-score) stat.

Table 4, extracted from TREC 2005 Overview, shows a comparison against other works of the conference. As seen, MLRBF offers trusted results with high precision (0.9102) on allele and 0.780 on GoA.

Several solutions on TREC were developed as a statistical system based on a semisupervised learning and modulating the original dataset under the medline mesh domain. Best results for other authors were achieved only if the mesh domain is used and no other data transformation is considered. Otherwise, applying other domains and building balanced systems, their results were similar to our system.

In conclusion, MLRBF may offer good results on general scenarios, even though a specific term domain is not specified.

## 5. Conclusions and Future Work

In this research, we present a new multikernel for SVM classifiers. The model divides the dataset in small portions to assign an independent kernel which is adjusted to take into account the containment of its slice.

The multikernel offers a stable behavior, thus avoiding some difficulties from text datasets. It achieved the best average results compared to other classifiers and some peaks as maximum values on precision or *F*-measure on allele corpus. Regarding GoA, our model got the best results in almost all statistics except for recall, in which the RBF kernel got better results keeping aside other stats.

With regard to runtime cost, the multikernel approach obtains a steady growth curve similar to linear kernel. Even so, MLRBF needs less time to completely build the model than RBF or Sigmoid.

At the end, we compare the novel model against other existing works in TREC 2005 competition, concluding that our system raises results under a high precision and *F*-measure stats even though a specific domain was not specified.

As future lines of work, we are going to focus our effort on reducing the computational cost of the preprocessing step when applying subsampling, which is known to produce different results because samples are not removed in the same order each time they are applied.

We are going to focus our effort on developing a preprocessing step which helps to avoid the quadratic programming cost, while at the same time solving the problem associated with applying the subsampling technique.

## Figures and Tables

**Algorithm 1:**
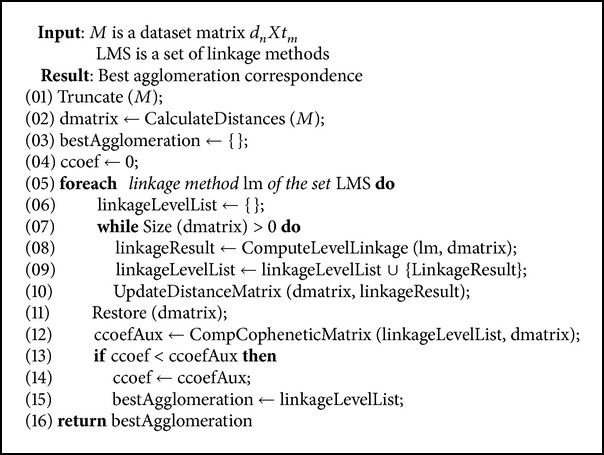
Hierarchical clustering algorithm (pseudocode).

**Algorithm 2:**
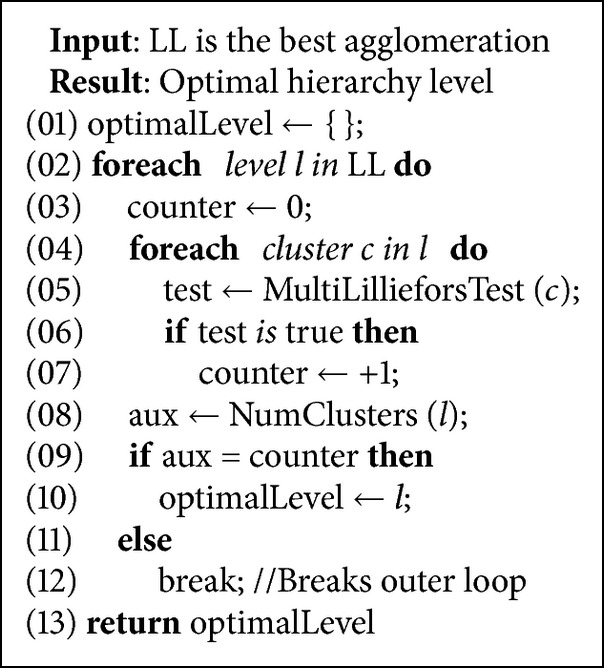
Optimal hierarchy level algorithm (pseudocode).

**Table 1:** Number of documents of the experimental corpora.

Corpus	Criteria					
TRAIN			TEST		
Total	Relevant	Nonrelevant	Total	Relevant	Nonrelevant

A (alelle)	5837	338	5499	6043	332
E (expression)	5837	81	5756	6043	105
G (GO annotation)	5837	462	5375	6043	518
T (tumor)	5837	36	5801	6043	20

**Table 2:** Comparative results between preconfigured classifiers and the proposed model.

Statistics	Classifiers			
Linear	RBF	Sigmoid	MLRBF

Allele			
Precision			
Min	0.840	0.645	0.830
Avg	0.891	0.725	0.855
Max	**0.913**	0.855	0.895
Recall			
Min	0.741	0.652	0.745
Avg	0.803	0.755	0.818
Max	0.834	0.834	**0.882**
*F*-measure			
Min	0.800	0.650	0.795
Avg	0.847	0.735	0.836
Max	0.872	0.844	**0.888**
Kappa			
Min	0.712	0.542	0.718
Avg	0.729	0.610	0.731
Max	0.754	0.692	**0.778**
GoA			
Precision			
Min	0.621	0.472	0.701
Avg	0.653	0.493	0.729
Max	0.689	0.501	0.759
Recall			
Min	0.584	0.496	0.619
Avg	0.617	0.640	0.662
Max	0.639	**0.797**	0.686
*F*-measure			
Min	0.602	0.490	0.657
Avg	0.635	0.552	0.694
Max	0.652	0.614	0.719
Kappa			
Min	0.227	−0.07	0.355
Avg	0.289	−0.01	0.416
Max	0.337	0.002	0.450

**Table 3:** Time (seconds) needed to build a model per number of folds.

Classifiers	Folds									
1	2	3	4	5	6	7	8	9	10

Allele									
Linear	4 s	9 s	13 s	17 s	21 s	26 s	30 s	34 s	39 s
MLRBF	15 s	31 s	46 s	62 s	77 s	93 s	108 s	124 s	139 s
Sigmoid	36 s	72 s	108 s	144 s	180 s	216 s	252 s	288 s	324 s
RBF	44 s	88 s	132 s	176 s	220 s	264 s	308 s	352 s	396 s
GoA									
Linear	7 s	14 s	21 s	28 s	35 s	42 s	49 s	56 s	63 s
MLRBF	35 s	70 s	105 s	140 s	175 s	210 s	245 s	280 s	315 s
Sigmoid	40 s	80 s	120 s	160 s	200 s	240 s	280 s	320 s	360 s
RBF	56 s	112 s	168 s	224 s	280 s	336 s	392 s	448 s	504 s

**Table 4:** Comparative against other authors (data source TREC 2005 [13]).

	Precision	Recall	*F*-score	Tag	Tag	*F*-score	Recall	Precision	
Allele	** 0.910 **	** 0.834 **	** 0.870 **	**MLRBF **	**MLRBF **	** 0.742 **	** 0.708 **	** 0.780 **	GoA
	0.541	0.867	0.666	THUIRgA0p9x	gibmadz05m1	0.423	0.617	0.321	
	0,507	0.900	0.649	aibmadz05m1	gibmadz05m2	0.420	0.621	0.317	
	0,502	0.900	0.645	aibmadz05m2	gibmadz05s	0.415	0.583	0.322	
	⋮	⋮	⋮	⋮	⋮	⋮	⋮	⋮	
	0.233	0.259	0.245	aLRIk1	gLRIk2	0.101	0.102	0.100	
	.0.230	0.250	0.239	aLRIk2	gMUSCUIUC2	0.100	0.173	0.070	
	0.219	0.262	0.238	aLRIk3	gLRIk1	0.097	0.102	0.093	

Min	0.219	0.250	0.238			0.097	0.102	0.070	Min
Avg	0.357	0.893	0.506			0.318	0.650	0.210	Avg
Max	0.795	0.957	0.666			0.423	0.936	0.554	Max

## References

[1] Salton G., Buckley C. (1988). Term-weighting approaches in automatic text retrieval. *Information Processing and Management*.

[2] Barandela R., Sanchez J. S., Garcia V., Rangel E. (2003). Strategies for learning in class imbalance problems. *Pattern Recognition*.

[3] Weiss G. M. (2004). Mining with rarity: a unifying framework. *ACM SIGKDD Explorations Newsletter*.

[4] Tan S. (2005). Neighbor-weighted K-nearest neighbor for unbalanced text corpus. *Expert Systems with Applications*.

[5] Borrajo L., Romero R., Iglesias E. L., Redondo Marey C. M. (2011). Improving imbalanced scientific text classification using sampling strategies and dictionaries. *Journal of Integrative Bioinformatics*.

[6] Kang P., Cho S. (2006). EUS SVMs: ensemble of under-sampled SVMs for data imbalance problems. *Neural Information Processing*.

[7] Romero R., Iglesias E. L., Borrajo L. (2011). Building biomedical text classifiers under sample selection bias. *International Symposium on Distributed Computing and Artificial Intelligence*.

[8] Romero R., Iglesias E. L., Borrajo L., Marey C. M. R. (2011). Using dictionaries for biomedical text classification. *Advances in Intelligent and Soft Computing*.

[9] Chang C.-C., Lin C.-J. (2011). LIBSVM: a Library for support vector machines. *ACM Transactions on Intelligent Systems and Technology*.

[10] Tseng K.-K., Li Y., Hsu C.-Y., Huang H.-N., Zhao M., Ding M. (2013). Computer-assisted system with multiple feature fused support vector machine for sperm morphology diagnosis. *BioMed Research International*.

[11] Pai T. W., Wang H. W., Lin Y. C., Chang H. T. (2011). Prediction of B-cell linear epitopes with a combination of support vector machine classification and amino acid propensity identification. *Journal of Biomedicine and Biotechnology*.

[12] Zhang W., Yoshida T., Tang X. (2008). Text classification based on multi-word with support vector machine. *Knowledge-Based Systems*.

[13] Hersh W., Cohen A., Yang J., Bhupatiraju R. T., Roberts P., Hearst M. TREC 2005 genomics track overview.

[14] Gönen M., Alpaydın E. (2011). Multiple kernel learning algorithms. *Journal of Machine Learning Research*.

[15] Cristianini N., Shawe-Taylor J. (2000). *An Introduction to Support Vector Machines: And Other Kernel-based Learning Methods*.

[16] Ben-Hur A., Noble W. S. (2005). Kernel methods for predicting protein-protein interactions. *Bioinformatics*.

[17] de Diego I. M., Moguerza J. M., Muñoz A., Roli F., Kittler J., Windeatt T. (2004). Combining kernel information for support vector classification. *Multiple Classifier Systems*.

[18] de Diego I. M., Muñoz A., Moguerza J. M. (2010). Methods for the combination of kernel matrices within a support vector framework. *Machine Learning*.

[19] Bach F. R., Lanckriet G. R. G., Jordan M. I. Multiple kernel learning, conic duality, and the SMO algorithm.

[20] Igel C., Glasmachers T., Mersch B., Pfeifer N., Meinicke P. (2007). Gradient-based optimization of kernel-target alignment for sequence kernels applied to bacterial gene start detection. *IEEE/ACM Transactions on Computational Biology and Bioinformatics*.

[21] Damoulas T., Girolami M. A. (2009). Pattern recognition with a Bayesian kernel combination machine. *Pattern Recognition Letters*.

[22] Girolami M., Rogers S. Hierarchic bayesian models for kernel learning.

[23] Bennett K. P., Momma M., Embrechts M. J. MARK: a boosting algorithm for heterogeneous kernel models.

[24] Bi J., Zhang T., Bennett K. P. Column-generation boosting methods for mixture of kernels.

[25] Lovins J. B. (1968). Development of a stemming algorithm. *Mechanical Translation and Computational Linguistics*.

[26] Porter M. F. (1980). An algorithm for suffix stripping. *Program*.

[27] Zhang J., Mani I. kNN approach to unbalanced data distributions: a case study involving information extraction.

[28] Zou Q., Wang Z., Guan X., Liu B., Wu Y., Lin Z. (2013). An approach for identifying cytokines based on a novel ensemble classifier. *BioMed Research International*.

[29] Estabrooks A., Jo T., Japkowicz N. (2004). A multiple resampling method for learning from imbalanced data sets. *Computational Intelligence*.

[30] Garner S. R. WEKA: the Waikato environment for knowledge analysis.

[31] Romero R., Iglesias E. L., Borrajo L. (2012). A comparative analysis of balancing techniques and attribute reduction algorithms. *6th International Conference on Practical Applications of Computational Biology & Bioinformatics*.

[32] Jolliffe I. T. (2002). *Principal Component Analysis*.

[33] Kim S., Rim H., Yook D., Lim H. Effective methods for improving Naïve Bayes text classifiers.

[34] Tang Y., Zhang Y.-Q., Chawla N. V. (2009). SVMs modeling for highly imbalanced classification. *IEEE Transactions on Systems, Man, and Cybernetics, Part B: Cybernetics*.

[35] Ali S., Smith K. A. Automatic parameter selection for polynomial kernel.

[36] Li C.-H., Ho H.-H., Liu Y.-L., Lin C.-T., Kuo B.-C., Taur J.-S. (2012). An automatic method for selecting the parameter of the normalized kernel function to support vector machines. *Journal of Information Science and Engineering*.

[37] Scholkopf B., Smola A. (2001). *Learning with Kernels: Support Vector Machines, Regularization, Optimization, and Beyond*.

[38] Hill T., Lewicki P. (2007). *Statistics, Methods and Applications*.

[39] Chang J. T., Raychaudhuri S., Altman R. B. Including biological literature improves homology search.

[40] Hsu C., Chang C., Lin C.

[41] Cunningham H., Wilks Y., Gaizauskas R. J. GATE—a general architecture for text engineering.

[42] Fisher D. H. (1987). Knowledge acquisition via incremental conceptual clustering. *Machine Learning*.

[43] Chernobai A., Rachev S., Fabozzi F. (2005). *Composite Goodness-of-Fit Tests for Left-Truncated Loss Samples*.

[44] Manly B. F. J. (1994). *Multivariate Statistical Methods: A Primer*.

[45] Arthur D., Vassilvitskii S. *k*-Means ++: The advantages of carefull seeding.

[46] Farris J. S. (1969). On the cophenetic correlation coefficient. *Systematic Zoology*.

[47] Witten I. H., Frank E. (2005). *Data Mining: Practical Machine Learning Tools and Techniques*.

